# Refining Drug-Induced Cholestasis Prediction: An Explainable Consensus Model Integrating Chemical and Biological Fingerprints

**DOI:** 10.1021/acs.jcim.4c02363

**Published:** 2025-05-27

**Authors:** Palle S. Helmke, Gerhard F. Ecker

**Affiliations:** Department of Pharmaceutical Sciences, 27258 University of Vienna, 1090 Vienna, Austria

## Abstract

Effective drug safety assessment, guided by the 3R principle (Replacement, Reduction, Refinement) to minimize animal testing, is critical in early drug development. Drug-induced liver injury (DILI), particularly drug-induced cholestasis (DIC), remains a major challenge. This study introduces a computational method for predicting DIC by integrating PubChem substructure fingerprints with biological data from liver-expressed targets and pathways, alongside nine hepatic transporter inhibition models. To address class imbalance in the public cholestasis data set, we employed undersampling, a technique that constructs a small and robust consensus model by evaluating distinct subsets. The most effective baseline model, which combined PubChem substructure fingerprints, pathway data and hepatic transporter inhibition predictions, achieved a Matthews correlation coefficient (MCC) of 0.29 and a sensitivity of 0.79, as validated through 10-fold cross-validation. Subsequently, target prediction using four publicly available tools was employed to enrich the sparse compound-target interaction matrix. Although this approach showed lower sensitivity compared to experimentally derived targets and pathways, it highlighted the value of incorporating specific systems biology related information. Feature importance analysis identified albumin as a potential target linked to cholestasis within our predictive model, suggesting a connection worth further investigation. By employing an expanded consensus model and applying probability range filtering, the refined method achieved an MCC of 0.38 and a sensitivity of 0.80, thereby enhancing decision-making confidence. This approach advances DIC prediction by integrating biological and chemical descriptors, offering a reliable and explainable model.

## Graphical abstract

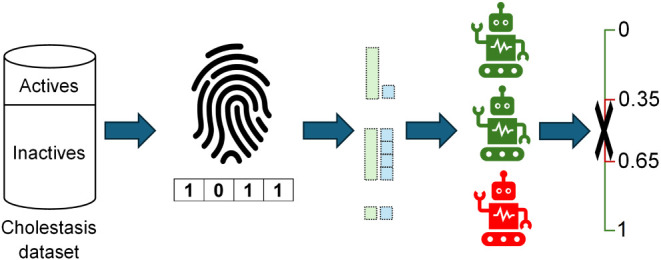

## Introduction

The safety assessment of drugs is a critical factor of early development stages, necessitating rigorous testing to ensure efficacy and minimize harm to future patients.^1^ Traditionally, animal testing has played a pivotal role in this process, providing essential data for toxicological assessment.^2^ However, ethical concerns surrounding animal welfare have prompted a reevaluation of these practices, leading to the adoption of the 3R principle—Replacement, Reduction, and Refinement.^3^ This dogma aims to mitigate ethical issues by minimizing animal use, enhancing experimental design, and seeking new approach methodologies (NAMs) where possible.^4^ In line with this shift, the FDA Modernization Act 2.0 has been introduced to further reduce reliance on animal testing by promoting the use of NAMs.^5^

A notable area of concern in drug safety is Drug-Induced Liver Injury (DILI), which remains a leading cause of drug withdrawal from the market.^6^ DILI can manifest as either an acute or chronic response to natural or synthetic compounds and is broadly categorized into intrinsic and idiosyncratic types.^7^ Intrinsic DILI is dose-dependent and predictable, whereas idiosyncratic DILI is unpredictable and not clearly dose-dependent.^8^ Idiosyncratic DILI can be further classified into hepatocellular, cholestatic, or mixed types based on liver enzyme patterns.^8^ Cholestatic DILI is characterized by the symptom of jaundice and constitutes more than 47% of all drug-related liver injuries.^9,10^ Nonsteroidal anti-inflammatory drugs (NSAIDs), antibiotics, statins, and anabolic agents are often responsible for cholestatic DILI cases.^10^ These drug classes vary in their structural characteristics as well as their cholestatic mode of action.

Three primary factors trigger drug-induced cholestasis (DIC), or more simply cholestasis. First, alterations in transporter function – such as direct inhibition, internalization, and reduced expression of basolateral and canalicular transporters play a crucial role.^11^ Examples of such transporters include the bile salt export pump (BSEP) and multidrug resistance protein 3 (MDR3), which are essential for hepatic clearance and bile salt secretion.^12,13^ Second, hepatocellular changes, including disruptions in cytoskeletal architecture, affect hepatocyte polarity through their impact on microtubules, cytokeratin intermediate filaments, tight junctions, and membrane fluidity. Third, alterations in bile canalicular dynamics, such as dilatation or constriction, disrupt bile contractile movement and removal. These factors trigger two cellular responses. (i) The adaptive response involves a coordinated interplay of bile acid and bilirubin-activated nuclear receptors, which work to counteract the (ii) deteriorative response, characterized by cellular injury mechanisms such as mitochondrial impairment and oxidative stress.^11,14^

The complexity of cholestasis mechanisms necessitates advanced modeling approaches, with in *vitro* models playing a key role. These models allow high-throughput screening of cholestatic drugs and provide mechanistic insights by linking cholestasis to the inhibition of transporters like BSEP,^15–17^ multidrug resistance-associated protein 3 (MRP3),^17,18^ and 4 (MRP4),^17,18^ and sodium (Na^+^) taurocholate cotransporting polypeptide (NTCP).^17,19^ However, in *vitro* models often focus on single parameters which may not fully capture the complexity of DIC. To address this limitation, integrating in *vitro* with in *silico* approaches is essential. In *silico* models, particularly those utilizing machine learning, enhance the prediction of hepatotoxicity by analyzing structural and physicochemical parameters, offering significant improvements in cost and time, while reducing animal testing.^11,12,20,21^

Building on these advancements, recent generalized DILI models leverage substructure information, such as molecular descriptors (InterDILI),^22^ Graph Neural Networks (GeoDILI),^23^ and structural similarity-based DILI occurrence analysis (Toxstar).^24^ To gain a mechanistic understanding of toxic events, it is crucial to integrate diverse data types, as is done in systems biology approaches.^25^ In this context, several methodologies have been developed for DILI prediction. Füzi et al.^26^ built tree-based binary classification models utilizing targets and pathways, while Shin et al.^27^ have created a unified network that integrates relevant genes and pathways.

Currently, there are few computational studies exclusively focused on DIC in the literature. Like in *vitro* approaches, some studies concentrate on predicting cholestasis through the modeling of hepatic transporter protein inhibition.^17,19^ This is partly due to the challenge of obtaining a sufficient data set including cholestasis-positive and -negative compounds. The existing real-world data shows a class imbalance, with a predominance of cholestasis inactive compounds, which complicates predictive modeling. To capture the complexity of cholestasis, different methodologies have been developed. For instance, Kotsampasakou and Ecker^12^ utilized transporter inhibition prediction models combined with physicochemical descriptors. Rodríguez-Belenguer et al.^28^ integrated various quantitative structure–activity relationship (QSAR) models with pharmacokinetic data through quantitative in *vitro* and in *vivo* extrapolation (QIVIVE). Additionally, Firman et al.^29^ developed structural alerts for the early identification of potential cholestatic effects, while Jiang et al.^30^ used machine learning and transcriptomic data to identify key gene signatures associated with DIC. These studies underscore the need for a deeper mechanistic understanding of cholestasis and highlight the importance of integrating chemical and biological information to enhance predictive models.

This study introduces a novel computational approach for modeling cholestasis, leveraging biological information and structural data. By integrating mechanistic and structural insights, our method enhances the explainability often missing in machine learning models used for toxicity prediction. Given the limited availability of experimental data on cholestasis in public databases, we employed four publicly available target prediction tools to enrich our model. Furthermore, to aid risk assessment and minimize uncertainty, we present a consensus cholestasis model and probability range filtering as additional measures.

## Methods

### KNIME

KNIME (Konstanz Information Miner) Analytics Platform (version 4.7.7) was utilized for data processing, machine learning and analysis in this study. This open-source software provides a robust environment for data-science purposes through an intuitive, node-based interface.^31^

### Data Set

The data set used in this study was originally collected by Kotsampasakou and Ecker^12^ and provided in a preprocessed format, where salts, inorganic compounds, and duplicates had already been removed. The initial data set comprised 1904 compounds, each with a binary categorization as cholestasis-positive or -negative. Using KNIME, ChEMBL IDs were successfully retrieved for 1722 of these compounds through drug names and IUPAC International Chemical Identifier Keys (InChIKeys), utilizing RDKit KNIME nodes (https://www.rdkit.org) and the ChEMBL Restful API (https://www.ebi.ac.uk/chembl/api/data/docs). The final data set consisted of 329 cholestatic and 1393 noncholestatic compounds. When available, drug names were used to retrieve ChEMBL IDs. Otherwise, InChIKeys generated from preprocessed SMILES were used. This ensured consistent classification and accurate mapping to cholestasis-associated compounds. Throughout this process and target prediction, stereochemistry was preserved. For calculating PubChem substructure fingerprints and modeling hepatic transporter inhibition, the data set was standardized with removed stereochemistry. Additionally, compounds were normalized, sanitized, reionized, uncharged and tautomers were canonicalized (https://github.com/sergsb/KNIME-standardizer). Sanitization refers to the process of correcting and standardizing molecular structures, such as ensuring proper valence, aromaticity, and tautomer normalization, thereby guaranteeing chemical validity. These are the standardization steps from our in-house pipeline for chemical fingerprint calculation used in modeling tasks.

### Biological Fingerprint: Compound-Target Bioactivity Data

Biological descriptors can be viewed as analogous to structural descriptors, where specific structural features are used to represent a compound. Likewise, a compound’s activity profile or signature is characterized by its presence or absence of activity across a panel of assays.^32,33^ For the generation of the biological fingerprints, the open-source KNIME workflow Path4Drug was utilized. The corresponding steps and filters are outlined in detail by Füzi et al.^34^ Biological fingerprints in this study consist of compound-target and compound-pathway fingerprints as well as the prediction of nine hepatic transporter inhibition models. In this study, an interaction between a compound and a biological target is defined as any activity data retrieved from one of the five databases: ChEMBL,^35^ DrugBank,^36^ PharmGKB,^37^ Therapeutic Target Database,^38^ and IUPHAR/BPS,^39^ using molecule ChEMBL IDs as input. The output was a binary matrix, where a value of one indicated an interaction between a compound and a target. In this KNIME workflow, human single protein targets were filtered and mapped to their corresponding Uniprot IDs. For ChEMBL, interactions were considered active if the reported pChEMBL value was ≥5, corresponding to an activity of ≤10 μM, a commonly used threshold for modeling tasks. pChEMBL is defined as −log_10_(molar IC50, XC50, EC50, AC50, *K*_i_, *K*_d_ or Potency), and is calculated and provided directly by the ChEMBL database.^40^ For the cholestasis data set, we retrieved pChEMBL values using the Path4Drug KNIME workflow, including the following standard types: IC50, EC50, *K*_i_, *K*_d_, AC50 and Potency. When multiple pChEMBL values were available for a given compound-target pair, the maximum value was selected to reflect the highest reported potency. Since pChEMBL values were binarized in the workflow, the exact numerical value was not critical if it was ≥5, in which case the interaction was considered active and assigned a value of one. The process of fingerprint retrieval is depicted in Figure 1.

**Figure 1:**
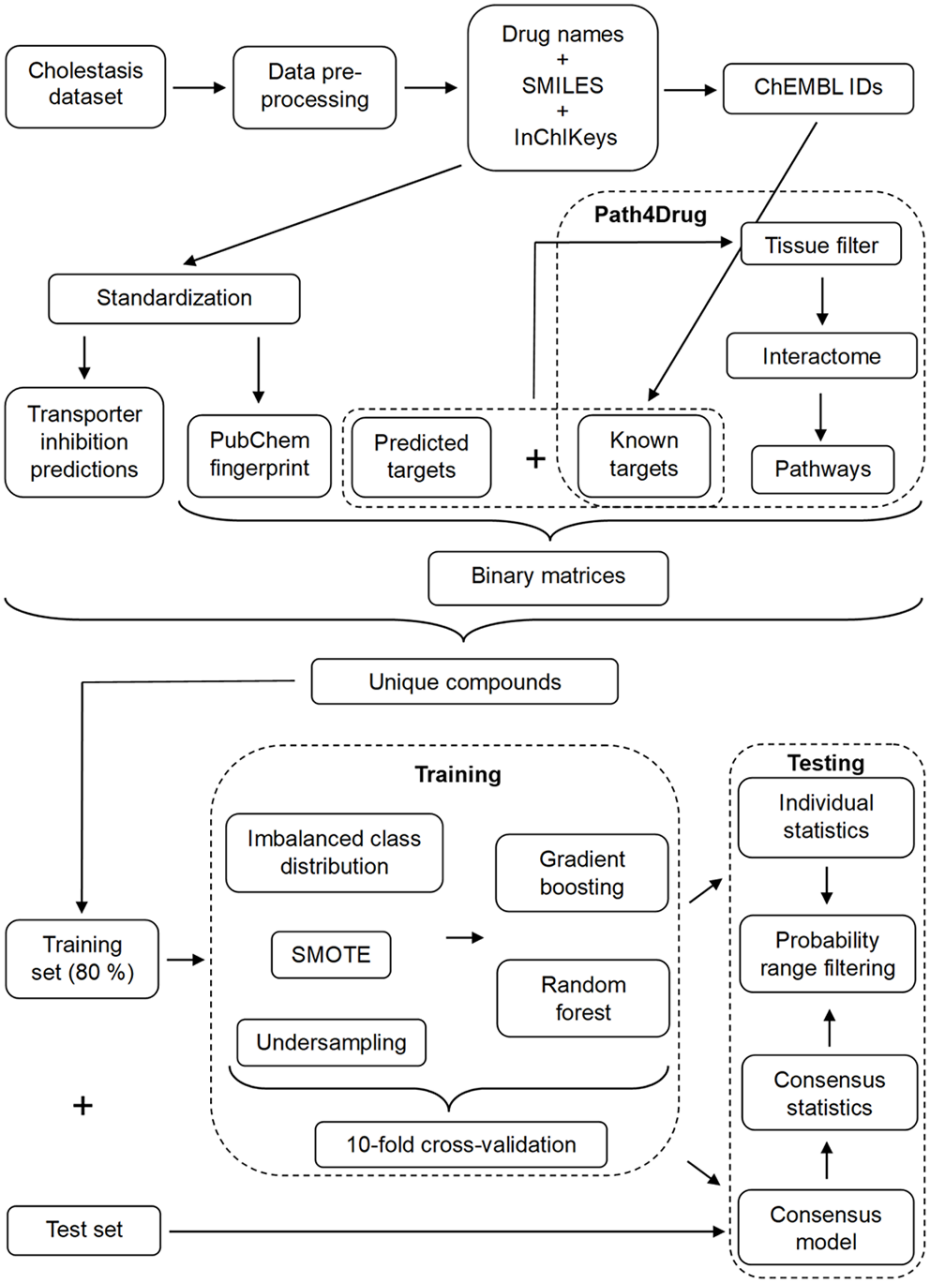
Depiction of the workflow used to generate the models in this study. SMILES were standardized for the generation of transporter inhibition models and PubChem fingerprints, which are steps of our in-house pipeline for chemical fingerprint calculation in modeling tasks. For the retrieval of ChEMBL IDs, drug names were used when available; otherwise, InChiKeys generated from preprocessed SMILES were utilized. Predicted and known targets were merged for the retrieval of pathway fingerprints in the retrained predicted target models. Subsequently, all fingerprints were converted into binary tables, that served as an input for the machine learning models.

### Biological Fingerprint: Predicted Compound-Target Interactions

To enrich the sparse bioactive compound-target interaction matrix, four publicly available target prediction tools were employed: Similarity Ensemble Approach (SEA)^41^ (https://sea.bkslab.org), TargetNet^42^ (http://targetnet.scbdd.com/), ChEMBL Mondrian Conformal Predictor^43^ (https://hub.docker.com/r/chembl/mcp) and ChEMBL Multitask Neural Network (MNN) (https://www.knime.com/blog/interactive-bioactivity-prediction-with-multitask-neural-networks).

### Consensus of Predicted Targets

In addition to incorporating all predicted compound-target interactions from the four prediction tools, a consensus approach was developed to improve the robustness of predicted targets. Separate KNIME workflows were established for the baseline model without target prediction and for each of the three cases including target prediction, to integrate unique predictions from all tools and consensus predictions shared by two or three tools. In the target prediction workflows, the predicted targets were merged with bioactive targets from Path4Drug, as outlined in Figure 1. The threshold criteria used to define predicted targets for the four tools are detailed in the Supporting Information (Table S1).

### Tissue Filter, Interactome, Liver-Expressed Pathways

The compound-target interactions, known and predicted, were further processed in Path4Drug by applying a tissue filter to focus on liver-expressed genes using The Human Protein Atlas.^44^ An interactome layer was then established to identify first degree interacting proteins of the direct targets, using data from the MINT^45^ and IntAct^46^ databases. Only human single protein targets were included. The identified interactors were grouped by compound-target interactions and assigned to pathways using the Reactome database^47^ (version 89). The pathways were subsequently compared to those expressed in the liver, as indicated by the Reactome tissue distribution tool (https://reactome.org/PathwayBrowser/#TOOL=AT).

### Transporter Inhibition Models

As another biological fingerprint, hepatic transporter inhibition profiles for breast cancer resistance protein (BCRP), bile salt export pump (BSEP), multidrug and toxin extrusion protein (MATE1), multidrug resistance protein 1 (MDR1), multidrug resistance-associated protein 3 (MRP3), organic anion transporting polypeptides 1B1 and 1B3 (OATP1B1 and OATP1B3), as well as organic cation transporter 1 and 2 (OCT1 and OCT2) were created using our in-house transporter model platform (Gaskin L and Ecker GF, manuscript in preparation). Transporter inhibition models were included based on publicly available data and their relevance as recognized by regulatory agencies, including the FDA, EMA and the Japanese regulatory agency. These authorities recommend, and in some cases mandate, routine inhibition and substrate studies for these transporters in the evaluation of new drugs.^48,49^ Briefly, five binary classification algorithms were trained using extended connectivity fingerprint 4 (ECFP4) for each transporter: logistic regression (LR), random forest (RF), k-nearest neighbors (kNN), and support vector regression (SVR) from Scikit Learn version 1.2.2, as well as extreme gradient boosted trees (XGB) from XGBoost version 1.7.4. However, for OATP1B1, the SVR algorithm was excluded due to performance issues. To ensure robust predictions a consensus strategy was employed: a compound was classified as an active inhibitor if at least three models predicted inhibition of the transporter. If fewer than three models predicted inhibition, the compound was classified inactive.

### PubChem Fingerprints

In addition to leveraging biological information, we investigated the potential relationship between molecular features and cholestasis by employing the PubChem fingerprint. This fingerprint provides detailed descriptions for 881 structural keys and allows for traceability in the feature importance analysis. Each bit in this fingerprint corresponds to the presence (1) or absence (0) of particular elements, ring systems, atom pairings, or atom environments (nearest neighbors) within the chemical structure.^50^ PubChem fingerprints were computed using the KNIME CDK Fingerprints node.^51^

### Binary Tables

After retrieving and calculating the fingerprints, binary tables were created for each fingerprint and their various combinations. In these tables, rows represented compounds, while columns denoted features such as targets, pathways, hepatic transporter inhibition predictions and PubChem bitvectors. A value of one indicated the presence of an interaction or feature, whereas a value of zero indicated its absence.

### Identification of Unique Compounds

In some cases, compounds with different PubChem fingerprints shared identical biological fingerprints. In other cases, compounds with conflicting class labels had identical biological fingerprints. Thus, we checked all compounds for duplicates in the fingerprint descriptor space separately for compound-target, compound-pathway, and PubChem fingerprints and used only those compounds in the final data set that were unique in all three fingerprints. This was essential to enable a fair comparison of model performances for models trained on individual fingerprints alone. The hepatic transporter inhibition predictions’ nine-bit fingerprint was excluded from this process.

### Calculation of Matrix Coverage

The biological fingerprint matrix coverage was calculated for compound-target and compound-pathway fingerprints. Specifically, to evaluate the coverage of cholestasis-related targets, we retrieved 420 genes associated with cholestasis from the DisGeNet^52^ (version 24.1) database and identified their presence in the compound-target interaction matrices of the baseline and target prediction models. The matrix coverages were calculated using the following formula:

Total possible interactions=compounds×targets

$$\mathrm{Matrix coverage}=\frac{\mathrm{Actual interactions}}{\mathrm{Total possible interactions}}$$

### Train/Test Split

The data sets containing unique compounds were divided into an 80% training set and a 20% holdout test set using stratified sampling based on the cholestasis classes. This approach allowed for consistent and unbiased performance evaluation, aligning with common practices in the field. Model validation was performed using 10-fold cross validation in concordance with FAIR principles as specified by Belfield et al.^53^ The best baseline model, as determined by cross-validation, was selected and retrained with the target prediction data sets. These were tested with the 20% holdout test set. To further assess the model’s generalizability beyond the training data, we validated it on an external test set composed of compounds without known compound-target interactions, thereby simulating a realistic scenario in which the model is applied to novel, uncharacterized compounds.

### H2O KNIME Framework

To assess the impact of individual features on cholestasis prediction, we employed the H2O framework within KNIME. H20.ai, accessible through the H2O Driverless AI extension in KNIME (https://h2o.ai/partner-network/find-a-partner/knime/), provides advanced automated machine learning solutions. In this study, we utilized RF and gradient boosted tree (GB) models from H2O.ai because of their capability to compute variable importance. The hyperparameters were set to the default KNIME settings, with detailed configurations and static random seeds as provided in the Supporting Information (Table S2). The output of the models was a probability score indicating whether a compound was classified as cholestasis-positive or not. A default probability threshold of 0.5 or higher indicated a positive classification and lower than 0.5 denoted a negative classification.

### Sampling Techniques

To address the class imbalance in the cholestasis data set, two techniques were applied. First, the Synthetic Minority Oversampling Technique (SMOTE) was implemented in KNIME, as originally proposed by Chawla et al.^54^ This method generates artificial minority class samples by interpolating between existing samples and their nearest neighbors to match the size of the majority class. In this study, five nearest neighbors were chosen. Second, undersampling (US) was used to reduce the majority class.^55^ For this, the majority class was divided into four subsets, each paired with the minority class to produce four model predictions per compound. Final classification was determined by consensus: if three or four models predicted cholestatic activity, the compound was classified as active; if none or only one predicted cholestatic activity, it was classified as inactive; for two active predictions, the mean probability was used. Class balancing techniques were applied only to the training set, including in 10-fold cross-validation, ensuring performance assessment on an unaltered test set.

### Performance Metrics

This study employed several statistical metrics to evaluate model performance, including accuracy, sensitivity, specificity, precision, the Matthews correlation coefficient (MCC), and balanced accuracy. The MCC is especially useful for assessing performance on imbalanced data sets, as it incorporates true positives (TP), false positives (FP), true negatives (TN), and false negatives (FN), thereby offering a more comprehensive measure than accuracy alone.^56^ This characteristic makes MCC a robust metric for evaluating binary classifications, particularly when class distributions are uneven.^57,58^ The MCC reads as follows:

$$\mathrm{MCC}=\frac{\mathrm{TP}\times \mathrm{TN}-\mathrm{FP}\times \mathrm{FN}}{\sqrt{\left(\mathrm{TP}+\mathrm{FP}\right)\left(\mathrm{TP}+\mathrm{FN}\right)\left(\mathrm{TN}+\mathrm{FP}\right)\left(\mathrm{TN}+\mathrm{FN}\right)}}$$

However, as balanced accuracy more effectively prioritizes models with adequate sensitivity and specificity over those with very high specificity but low sensitivity, it was chosen for model selection, making it more suitable for toxicity prediction tasks compared to MCC, which may favor imbalanced performance. The balanced accuracy is defined as follows:

$$\mathrm{balanced accuracy}=\frac{\mathrm{sensitivity}+\mathrm{specificity}}{2}$$

A detailed analysis is provided in the Supporting Information (Table S3).

### Feature Space Analysis

To assess differences in model performance across the baseline and three target prediction data sets, we used uniform manifold approximation and projection (UMAP)^59^ for clustering cholestasis-positive and cholestasis-negative compounds based on their feature vectors. UMAP, applying Euclidean distance for dimensionality reduction to two dimensions, was employed. For each data set, the combined feature vector, comprising targets, pathways, hepatic transporter inhibition predictions, and PubChem bitvectors, was utilized. The analysis was conducted using the Univie UMAP Jupyter Notebook (https://github.com/PharminfoVienna/Feature-space-analysis).

### Model Selection Process

After training six models for each individual and combined fingerprint using 10-fold cross-validation, the best baseline model was selected based on the balanced accuracy. It was chosen for this purpose, as it more effectively prioritizes models with adequate sensitivity and specificity over those with very high specificity but low sensitivity, making it more suitable for toxicity prediction tasks than MCC, which may favor imbalanced performance (Table S3). This model was then retrained with the target prediction tool data sets. Since the two models, trained on undersampled data, each included four sub models, a total of 12 models were trained for each data set. From these, the top nine models were chosen for the expanded consensus approach. Details of the model selection process are described in Figure 2.

**Figure 2:**
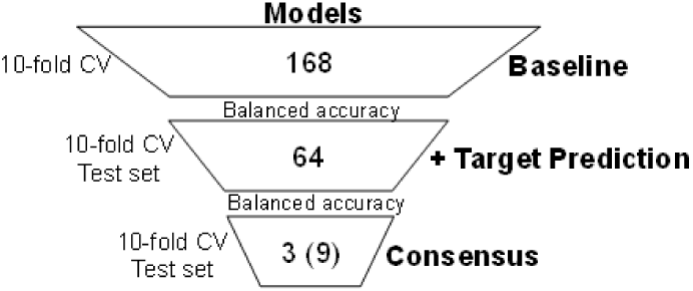
Model selection according to the highest balanced accuracy.

### Consensus Models

A consensus-based approach was established, combining GB and RF models with SMOTE and undersampling to address class imbalance. Models trained on the imbalanced data set were excluded from the consensus, and three models were selected by discarding the one with the lowest balanced accuracy for majority voting. With two models trained on undersampled data, each using four subsets, a total of nine models were generated. Compounds were classified as active if predicted as such by at least five models; otherwise, they were inactive. Model and consensus-building details are shown in Figure 3.

**Figure 3:**
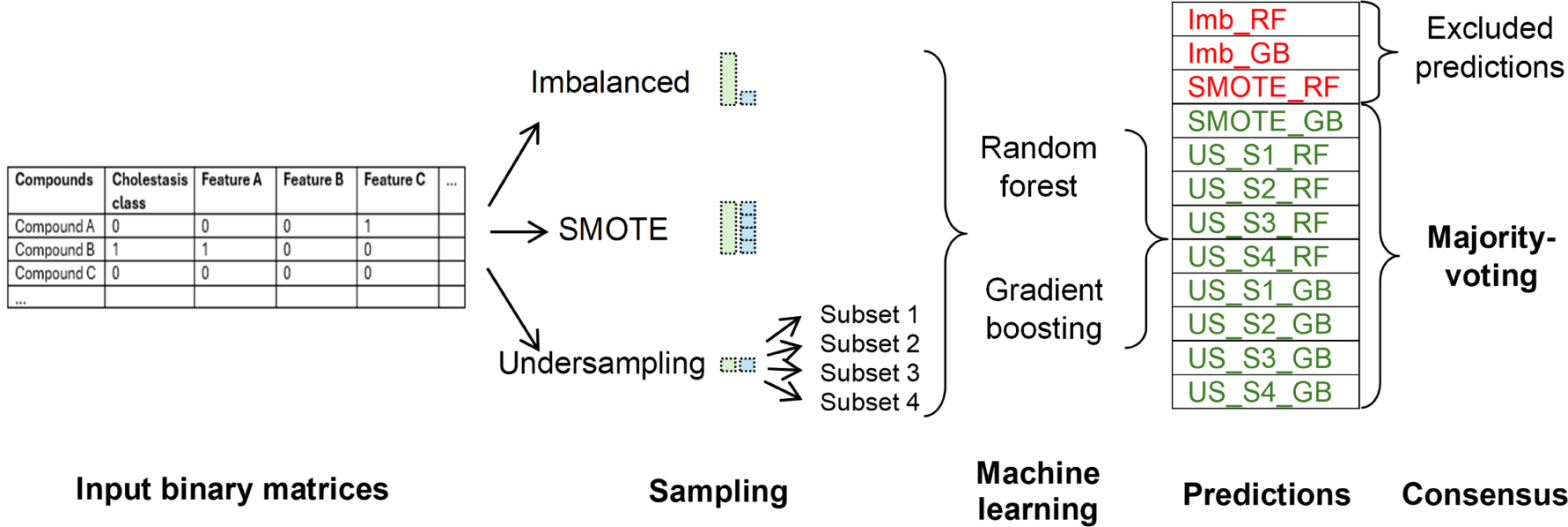
Individual and consensus model building.

### Probability Range Filtering

To enhance the reliability of cholestasis predictions, probability range filtering was applied to both the consensus model and the top-performing individual model. Predictions with probabilities between 0.35 and 0.65 were excluded to focus on predictions with higher confidence. For the individual model, which comprises four sub models, and the extended consensus model, consisting of nine sub models, the mean probability was used for filtering.

## Results

### Compound Data Set

The experimental biological data from Path4Drug along with nine hepatic transporter inhibition predictions and PubChem bitvectors provided the baseline models. To expand on this and enrich the sparse bioactive compound-target interaction matrix, three target prediction data sets were developed: one incorporating predictions from all tools (+all), one based on the consensus from two tools (+2-C), and one derived from consensus predictions from three tools (+3-C). In these data sets, unique compounds with respect to each fingerprint were identified, and only unique compounds common across all fingerprints were merged to create the final data sets. As can be seen in Table 1, incorporating predicted targets impacted both the size of the data sets and their class balance. Specifically, the +all data set contained the largest number of compounds, but it also showed the highest class imbalance due to a predominant enrichment of noncholestatic compounds.

**Table 1:** Data Set Sizes and Class Balance of Baseline and Predicted Target Models

data set	no. of unique compounds	class balance	Chol.–	Chol.+	training set (80%)	test set (20%)
Baseline	899	3.26:1	688	211	719	180
+all	1655	4.29:1	1342	313	1324	331
+2-C	1214	3.74:1	958	256	971	243
+3-C	906	3.31:1	696	210	724	182

### Fingerprint Sizes

The number of liver-expressed targets differed across the data sets (Table 2) with additional predicted target data sets including a higher number of targets and consequently more compound-target interactions. Since pathways are connected through targets and their first level interactome, this trend similarly affected the number of pathways and compound-pathway interactions. As shown in Table 2, the +all data set had the highest number of features and interactions in all fingerprints. The number of interactions in the PubChem fingerprint and hepatic transporter Inhibition models increased with data set size, but their bit-length remained constant. Interestingly, the number of cholestasis-related genes from DisGeNet, that were solely used for analysis and not as a fingerprint for model training, only varies slightly between the baseline and target prediction models.

**Table 2:** Number of Compound-Interactions per Fingerprint and Corresponding Bit Sizes (Unique Features)^a^

	baseline		+all		+2-C		+3-C	
fingerprint	unique features	cmpd. interactions	unique features	cmpd. interactions	unique features	cmpd. interactions	unique features	cmpd. interactions
Liver-expressed targets	1,079	8,603	1,243	92,919	1,105	11,659	1,082	8,667
Liver-expressed pathways	2,051	210,054	2,065	1,817,211	2,052	327,628	2,051	211,908
PubChem	881	114,808	881	202,326	881	152,529	881	115,680
Transporter inhibition models	9	565	9	853	9	695	9	569
Cholestasis-related genes	117	3,713	124	10,902	122	4,157	117	3,718

a In addition, the number of cholestasis-related genes from DisGeNet in each data set are shown.

Figure 4 presents the fingerprint length of the baseline and target prediction models in comparison to the maximum fingerprint length. This maximum length was defined by the total number of liver-expressed targets identified using the Path4Drug tissue filter, liver-expressed pathways obtained through the Reactome tissue distribution tool, PubChem bitvectors and nine transporter inhibition predictions, which are not shown in the figure for visualization purposes. Notably, although the fingerprint length considering only unique features varied slightly between the models, the number of interactions significantly increased when predicted targets were included (Table 2).

**Figure 4:**
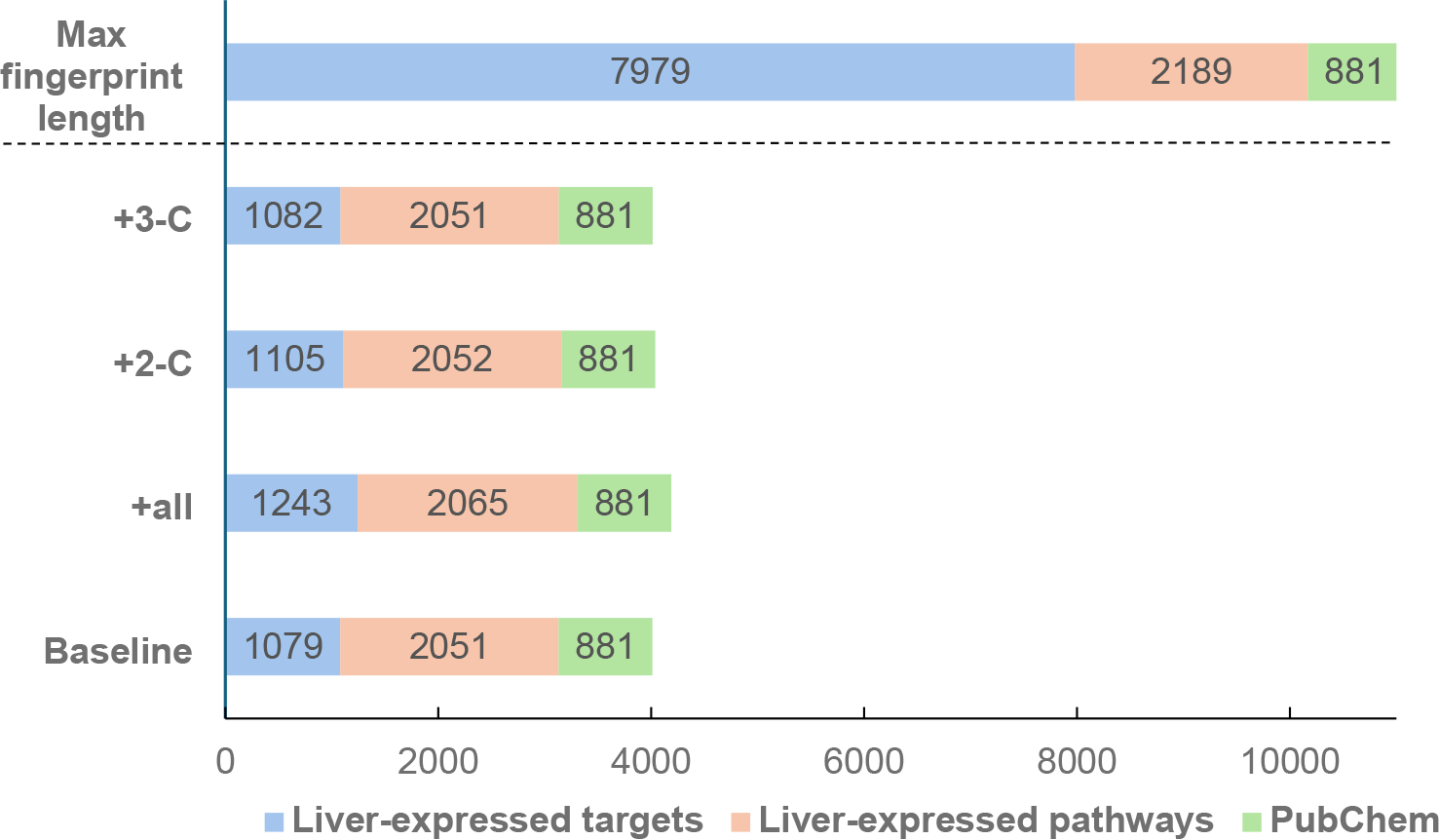
Illustration of the fingerprint length for both the baseline and target prediction models in comparison to the maximum attainable fingerprint length.

### Matrix Coverage of Biological Fingerprints and Cholestasis-Related Targets

The matrix coverages of liver-expressed targets across the models were low, with the +all data set showing the highest with 4.52%, as shown in Table 3. Interestingly, the coverages of cholestasis-related genes only varied slightly between baseline and enriched models. In contrast, liver-expressed pathways had significantly higher matrix coverages across all models, with the highest observed in the +all data set (Table 3).

**Table 3:** Matrix Coverages of Biological Compound-Fingerprint Matrices

fingerprint	baseline	+all	+2-C	+3-C
Liver-expressed targets	0.89%	4.52%	0.87%	0.88%
Cholestasis-related targets	3.53%	5.31%	2.81%	3.51%
Liver-expressed pathways	11.39%	53.17%	13.15%	11.40%

### Model Performances of Baseline Models and Selection of Best Models

Different sets of descriptors were used to train RF and GB models. Given the imbalanced nature of the cholestasis data set, both tree-based algorithms were paired with different sampling techniques to improve model performance. This section of the results provides a summary of the statistical evaluation of models generated from distinct fingerprints and their combinations. The baseline models were developed using 1,079 liver-expressed targets, 2,051 liver-expressed pathways, 881 bits from the PubChem fingerprint, and nine hepatic transporter inhibition prediction models (Table 2). Only the best baseline model per fingerprint is shown. The detailed performance statistics for the baseline models are provided in the Supporting Information (Table S3).

Models trained with undersampled data (US), implemented through four sub models, consistently outperformed those trained using SMOTE and the imbalanced data sets across all fingerprints (Table S3), showing superior performance in eight cases with the RF algorithm and in six cases with the GB algorithm (Table 4). To determine the optimal model for retraining with predicted targets, the balanced accuracy was used. The combined model that integrates PubChem fingerprints, pathway information, and hepatic transporter inhibition predictions demonstrated superior performance compared to individual and other combined fingerprints. It achieved the highest sensitivity and a balanced accuracy of 0.67, indicating the best overall performance for a toxicity prediction task when evaluated to the third decimal place (Table S3).

**Table 4:** Model Performance of the Best Baseline Models for Individual and Combined Fingerprints with 10-Fold Cross-Validation

fingerprint	best model settings	accuracy	MCC	sensitivity	specificity	precision	balanced accuracy
PubChem	US_GB	0.57	0.23	0.76	0.52	0.33	0.64
Target	US_RF	0.60	0.26	0.75	0.56	0.34	0.65
Pathway	US_RF	0.56	0.22	0.76	0.50	0.32	0.63
PubChem + Target	US_GB	0.62	0.24	0.69	0.59	0.34	0.64
PubChem + Pathway	US_GB	0.61	0.29	0.79	0.55	0.35	0.67
Target + Pathway	US_RF	0.56	0.22	0.75	0.50	0.32	0.63
PubChem + Transporter	US_RF	0.54	0.22	0.79	0.46	0.31	0.63
Target + Transporter	US_RF	0.59	0.25	0.75	0.54	0.33	0.65
Pathway + Transporter	US_RF	0.57	0.24	0.78	0.50	0.33	0.64
Target + Pathway + Transporter	US_RF	0.57	0.24	0.76	0.51	0.33	0.64
PubChem + Target + Pathway	US_GB	0.61	0.27	0.76	0.56	0.35	0.66
PubChem + Target + Transporter	US_RF	0.58	0.24	0.75	0.53	0.33	0.64
**PubChem + Pathway + Transporter**	**US_GB**	**0.61**	**0.29**	**0.79**	**0.55**	**0.35**	**0.67**
PubChem + Target + Pathway + Transporter	US_GB	0.61	0.26	0.73	0.57	0.35	0.65

### Retraining with Predicted Targets

Retraining the top-performing baseline model with all predicted targets and consensus target prediction approaches led to a reduced sensitivity during 10-fold cross-validation across all predicted target models compared to baseline. The +all model showed a slight increase in MCC during cross-validation, mainly due to enhanced specificity but reduced sensitivity (Table 5). The best individual model performance was achieved in all cases with models trained on undersampled data using the GB algorithm. The detailed performance statistics for the retrained target prediction models are presented in the Supporting Information (Table S4).

**Table 5:** Best Model Performance of Retrained Target Prediction Models in Comparison to the Best Baseline Model for the Combined PubChem/Pathway/Transporter Fingerprint^a^

	accuracy		MCC		sensitivity		specificity		precision		balanced accuracy	
model	CV	test	CV	test	CV	test	CV	test	CV	test	CV	test
Baseline	0.61	0.64	0.29	0.31	0.79	0.76	0.55	0.61	0.35	0.37	0.67	0.69
+all	0.72	0.70	0.30	0.27	0.62	0.59	0.74	0.73	0.36	0.34	0.68	0.66
+2-C	0.66	0.62	0.28	0.24	0.69	0.69	0.65	0.60	0.35	0.32	0.67	0.65
+3-C	0.62	0.59	0.25	0.26	0.70	0.76	0.60	0.54	0.34	0.33	0.65	0.65

a The best model performance was achieved in all cases combining US and the GB model.

### Feature Space

The feature spaces of the cholestasis data sets were separately analyzed using UMAP to explain differences in model performance. Each compound in the data sets was represented by the combined PubChem bitvector, compound-target and compound-pathway fingerprints, as well as the nine hepatic transporter inhibition predictions. Cholestasis-positive compounds were marked red, and cholestasis-negative compounds green (Figure 5).

**Figure 5:**
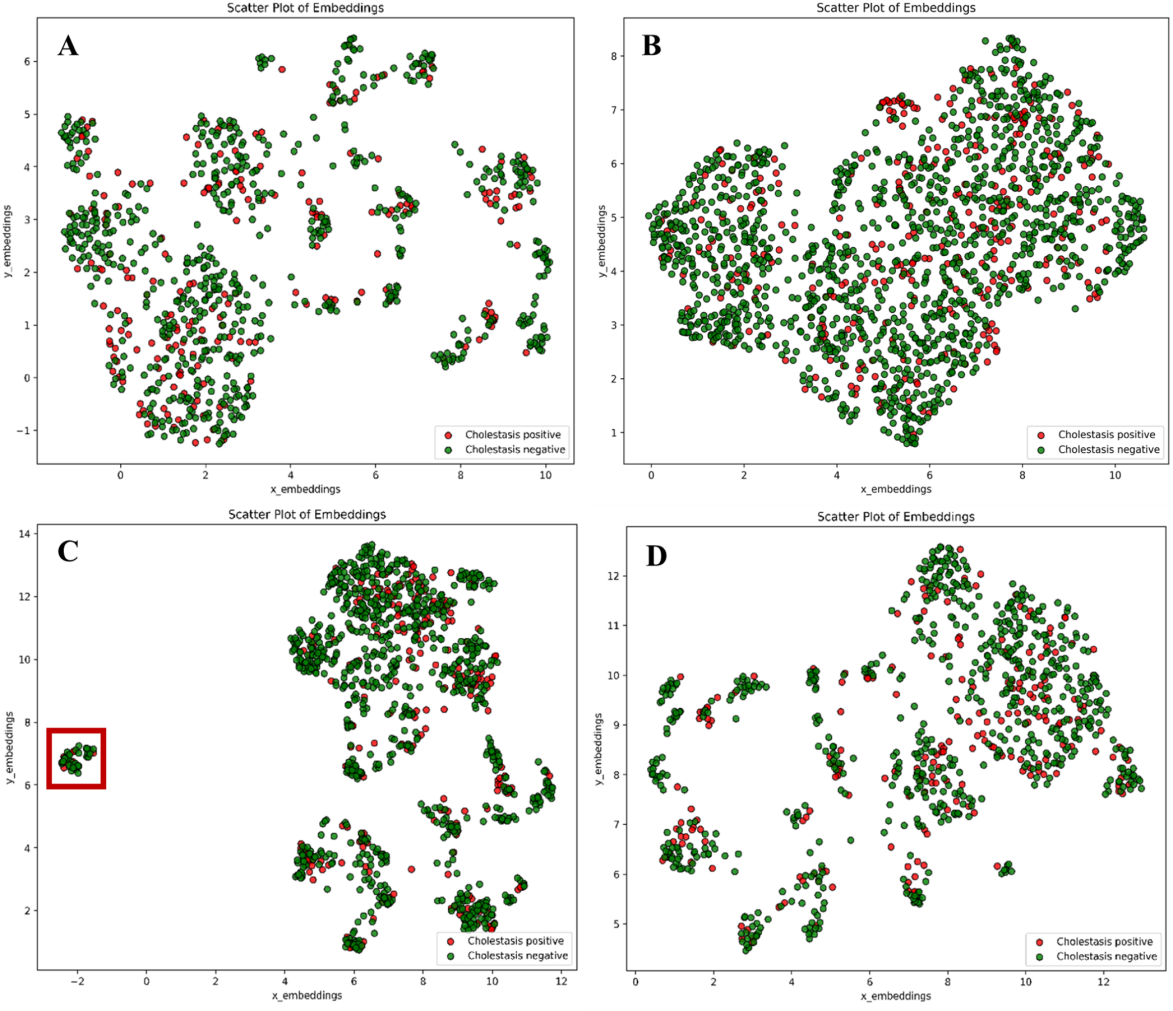
Visualization of the feature space of the baseline, +all, +2-C and +3-C models combining all features. (A) Feature space of the baseline data set. (B) +all. (C) +2-C. (D) +3-C. Green indicates cholestasis-negative compounds, red indicates cholestasis-positive compounds.

No distinct grouping was evident in all models between cholestasis-positive and cholestasis-negative compounds, which can be seen in Figure 5. However, in the baseline (Figure 5A), the +2-C (Figure 5C) and the +3-C (Figure 5D) models, some grouping in the feature space was observed. This also accounted for cholestasis-positive compounds forming some groups in between cholestasis-negative compounds. These groups were lost when including all targets (Figure 5B), with cholestasis-positive compounds broadly dispersed in the feature space. Thus, the inclusion of more cholestasis-negative compounds and additional features through target prediction impacted both general clustering behavior and the grouping of cholestasis-positive compounds. Notably, the red highlighted region in Figure 5C marks a cluster of 49 compounds within the feature space of the +2-C model. These compounds all interact with the Amyloid-beta precursor protein, which is connected to 347 liver-expressed pathways through its interactome. As a result, these compounds share these features of the fingerprint.

### Consensus Modeling

To enhance confidence in cholestasis prediction, a consensus model was developed. The baseline model, which outperformed the target prediction models, was utilized for this approach. Initially, six models were trained for each fingerprint (Figure 3). However, models trained on imbalanced data sets were excluded due to their high specificities and low sensitivities (Table S3), which skewed outcomes toward inactivity. An odd number of models was chosen to reduce prediction ambiguity. Consequently, the final consensus approach incorporated nine models: two models trained on undersampled data, each with four subsets, and the top-performing model using SMOTE on the training set.

Compared to the best individual baseline model (Table 4), the MCC, sensitivity and balanced accuracy of the consensus approach decreased slightly in 10-fold cross-validation and the test set, while specificity slightly increased in 10-fold cross-validation, as depicted in Table 6.

**Table 6:** Model Performance of the Consensus Approach of the Best Baseline Model

	accuracy		MCC		sensitivity		specificity		precision		balanced accuracy	
model	CV	test	CV	test	CV	test	CV	test	CV	test	CV	test
Baseline	0.61	0.62	0.25	0.26	0.73	0.71	0.57	0.59	0.34	0.35	0.65	0.65

### Probability Range Filtering

To further reduce uncertainty in cholestasis prediction, probability range filtering was applied, excluding uncertain predictions within the 0.35 to 0.65 probability range. This approach improved all performance metrics for the top-performing individual baseline model (Table 4) and the consensus baseline model (Table 6), as indicated by 10-fold cross-validation and test set results (Table 8). The observed differences in MCC and sensitivity between 10-fold cross-validation and the test set in the individual model can be explained by the effect of probability range filtering. Specifically, excluding cholestasis-positive compounds with prediction probabilities in the uncertain range (0.35–0.65) led to a markedly lower proportion of false negatives in the test set compared to the training set (Table 7). This resulted in a substantial increase in both MCC and sensitivity, as shown in Table 8. The increase in all performance metrics was higher for the consensus model.

**Table 7:** Distribution of Compounds across Probability Ranges for the Cholestasis Classes in 10-Fold Cross-Validation and the Independent Test Set

	train		test	
probability range (US_GB)	Chol.–	Chol.+	Chol.–	Chol.+
0–0.35	224	28	57	4
0.35–0.65	156	36	43	10
0.65–1	170	105	38	28

**Table 8:** Model Performance of Probability Range Filtering for the Best Individual Model and the Consensus Model

	accuracy		MCC		sensitivity		specificity		precision		balanced accuracy	
model	CV	test	CV	test	CV	test	CV	test	CV	test	CV	test
US_GB	0.62	0.67	0.31	0.41	0.79	0.88	0.57	0.60	0.38	0.42	0.68	0.74
Consensus	0.68	0.73	0.38	0.46	0.80	0.84	0.64	0.69	0.42	0.48	0.72	0.77

In both the 10-fold cross-validation and the test set, the consensus approach excluded more compounds compared to the individual baseline model, as can be seen in Table 9. Across all models, the ratio of cholestasis-negative to cholestasis-positive compounds among the excluded compounds was slightly higher than the original class balance of 3.26:1 of the baseline model (Table 1). This suggests that more cholestasis-negative compounds were classified within the uncertain prediction range between 0.35 and 0.65. The class balance of the excluded compounds in the consensus model was closer to the original baseline data set than in the model trained on undersampled data. This indicates that the consensus model more effectively excluded compounds from both cholestasis classes, aligning with its higher increase in specificity compared to the baseline model.

**Table 9:** Number of Excluded Compounds in the Probability Range Filtering Approach That Fall in the Uncertain 0.35 to 0.65 Probability Range

	train (719 compounds)				test (180 compounds)			
model	excluded compounds	Chol.–	Chol.+	class balance	excluded compounds	Chol.–	Chol.+	class balance
US_GB	192	156	36	4.33:1	53	43	10	4.30:1
Consensus	311	243	68	3.57:1	81	64	17	3.76:1

### Compound Prioritization

To evaluate model usability for compound prioritization, 421 compounds without known liver-expressed targets were selected from the original data set including 54 cholestasis-positive and 367 cholestasis-negative compounds. This represents a use case in which only the molecular structure of compounds is available as a SMILES annotation. Liver-expressed targets for these compounds were predicted using the four target prediction tools, with all predicted liver-expressed targets included for further processing as outlined in Figure 1. The best performing baseline model, integrating liver-expressed pathways, PubChem bitvectors, and transporter inhibition predictions, was then applied to predict cholestatic activity for these compounds. Notably, the MCC decreases, as shown in Table 10, compared to the best-performing baseline model (Table 4), while sensitivity remains stable at 0.82.

**Table 10:** Model Performance of Compounds without Known Liver-Expressed Targets for Compound Prioritization

model	accuracy	MCC	sensitivity	specificity	precision	balanced accuracy
Baseline	0.51	0.19	0.82	0.47	0.18	0.65

### Combined Fingerprint Descriptor Importance of the Baseline Model

Assessing the impact of all features on model performance, the best individual model was selected (undersampling combined with the GB algorithm), along with integrated fingerprints from PubChem, targets, pathways, and hepatic transporter inhibition predictions. Given that undersampling involved four sub models, the mean scaled influence of all features was calculated and ranked in descending order. The top 10 features, detailed in Table 11, included four pathways, four PubChem bitvectors and two targets. Among the top 24 features, which had a mean scaled influence of 0.1 or higher, there were 13 pathways, four targets, and seven PubChem bitvectors. Interestingly, no hepatic transporter inhibition predictions were found in the top descriptors even though they were in the combined descriptor set yielding the best performance for the baseline models. The best ranked transporter inhibition prediction model was OCT2 on position 121 with a mean scaled influence of 0.036.

**Table 11:** Top 10 Descriptors of the Combined Descriptor Set of the Best Baseline Model with the 20% Holdout Test Set

fingerprint identifier	type	description	Chol.+	ratio in + class	Chol.–	ratio in – class
R-HSA-2142753	Pathway	Arachidonic acid metabolism	146	0.69	320	0.47
R-HSA-1430728	Pathway	Metabolism	116	0.55	225	0.33
P02768	Target	Albumin	85	0.40	151	0.22
R-HSA-8978868	Pathway	Fatty acid metabolism	120	0.57	233	0.34
O95342	Target	Bile Salt Export Pump	45	0.21	53	0.08
Bitvector797	PubChem	CC1CC(C)CCC1	32	0.15	156	0.23
Bitvector439	PubChem	C(−C)(−N)(=O)	60	0.28	165	0.24
R-HSA-1660661	Pathway	Sphingolipid de novo biosynthesis	56	0.27	96	0.14
Bitvector353	PubChem	C(∼C)(∼S)	63	0.30	104	0.15
Bitvector590	PubChem	C–C:C–O–[#1]	25	0.12	99	0.14

In the baseline data set, comprising 211 cholestasis-positive and 688 cholestasis-negative compounds (Table 1), the ratio of compounds interacting with the top descriptors is consistently higher in the cholestasis-positive class with the exception of PubChem bitvectors 797 and 590. This indicates that aside from these two bitvectors, the descriptors generally provide a stronger signal for the cholestasis-positive class. Table 12 presents selected cholestasis-positive compounds that contain the highly ranked PubChem substructures.

**Table 12:**
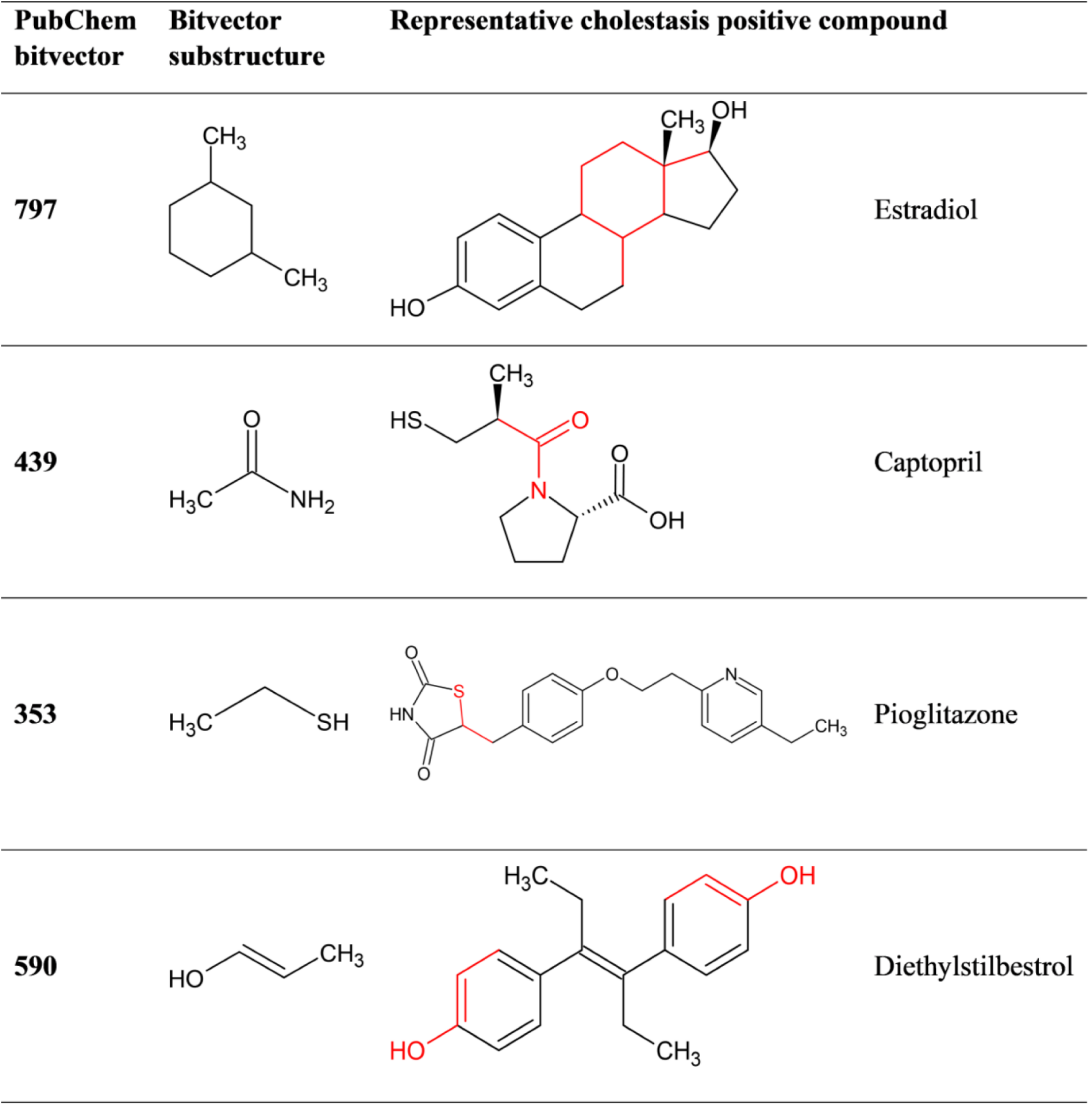
Representative Cholestasis-Positive Compounds with Top-Ranked PubChem Substructures Identified in the Feature Importance Analysis

### Albumin Interaction

Albumin, the most prevalent plasma protein, was identified as the third ranked feature of the model combining all fingerprints (Table 11). Despite this, its role in DIC remains underexplored. To investigate a potential link, Drug Bank pharmacology information was utilized.^36^ Albumin-interacting compounds from the cholestasis and DILI rank data sets were manually classified from the literature into four plasma protein binding categories: high (≥90% or term like “highly plasma protein bound”), medium (60–90%), low (<60%) binding, and inconclusive or no evidence. When a protein binding term encompassed a range spanning two categories, the lower category was assigned for classification. Additionally, metabolite data was not considered for the analysis. Specifically, 98 compounds from the cholestasis-positive and 179 from the cholestasis-negative class interacted with albumin, considering the entire cholestasis data set of 1904 compounds. This indicates a higher proportion of albumin interacting compounds in the cholestasis-positive class, given the class imbalance. Further analysis revealed that a higher proportion of compounds in the cholestasis-positive class interacting with albumin, were categorized in the high plasma protein binding group compared to those in the cholestasis-negative class, as shown in Figure 6. This trend was also observed in the DILI rank data set. This data set was provided by the FDA and categorizes drugs into four classes based on their risk to cause DILI: most-DILI, less-DILI, ambiguous-DILI and no-DILI concern.^60^ Among the 1,036 compounds of the DILI rank data set, 395 were classified as either most-DILI or less-DILI concern, collectively termed as DILI-positive for the plasma protein binding analysis, while 223 compounds were categorized as no-DILI concern, with 254 ambiguous cases excluded. 164 compounds from the DILI rank data set were excluded due to missing PubChem CIDs or mapping to ChEMBL IDs. As shown in Table 13, 119 compounds in the DILI-positive class interacted with albumin, compared to 29 compounds in the no-DILI concern class, indicating a prevalence of albumin interacting compounds in the DILI-positive class. Similarly to the cholestasis-positive compounds, DILI-positive compounds were more frequently associated with high plasma protein binding than compounds of the no-DILI concern class (Figure 6). Conversely, fewer cholestasis-positive and DILI-positive compounds with albumin interaction were found in the low and medium plasma protein binding categories.

**Table 13:** Albumin Interactions of Compounds in the Cholestasis and DILI Rank Datasets

data set	compounds	interacting with albumin in + class	interacting with albumin in – class
Cholestasis	1904	98	179
DILI rank	1036	119	29

**Figure 6:**
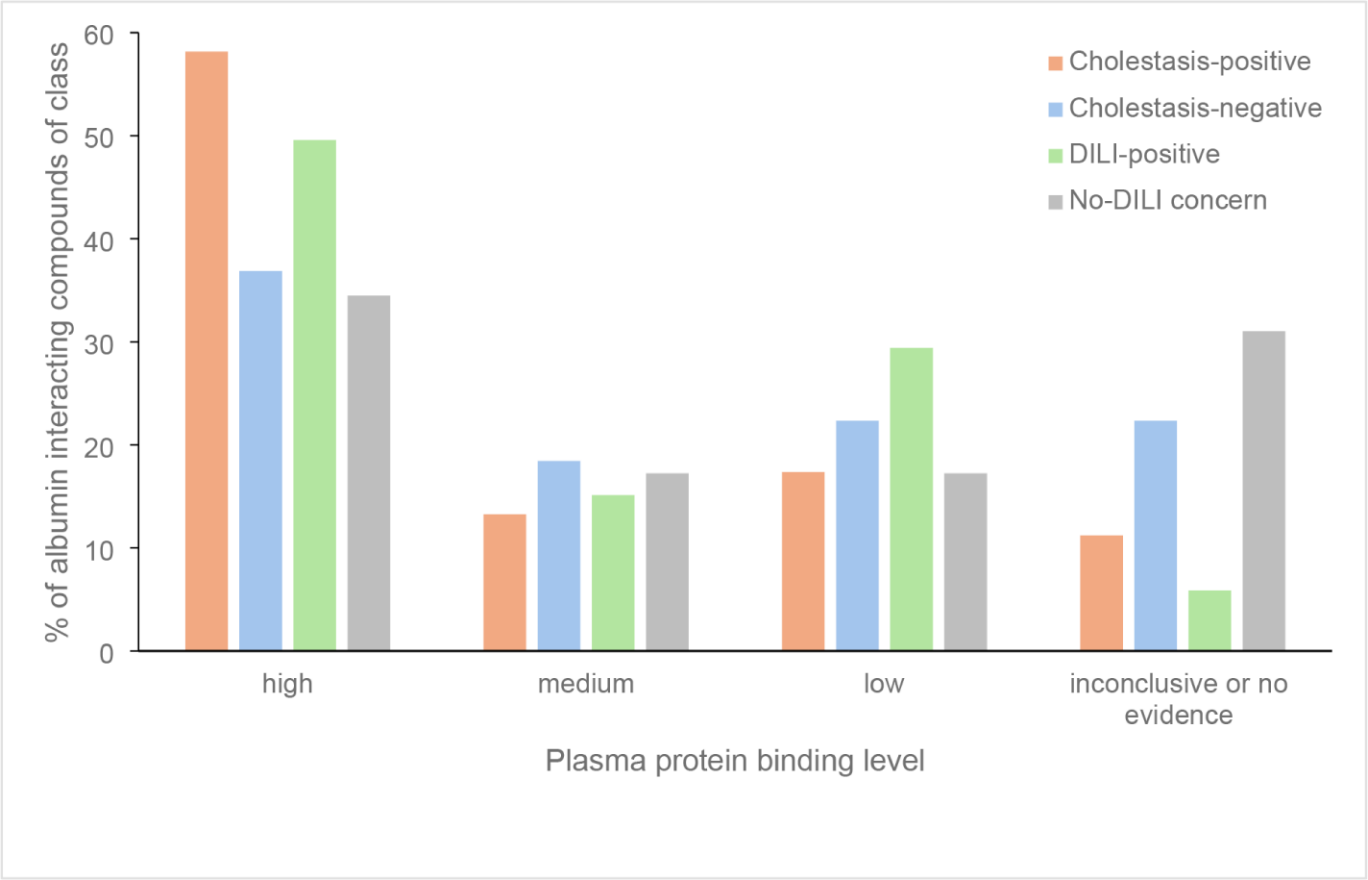
Bar chart showing percent of albumin interacting compounds in the cholestasis and DILI rank data sets in high, medium, low and inconclusive or no evidence plasma protein binding level categories.

## Discussion

Predicting DIC as a clinical manifestation of DILI, along with providing a mechanistic explanation for the prediction, is a challenging endeavor. This study aimed to address this complexity by integrating traceable chemical information such as PubChem fingerprints, with biological data, including target and pathway fingerprints and hepatic transporter inhibition predictions. This approach prompted further questions about whether enriching the compound-target interaction matrix could improve model performance and whether employing a consensus approach and probability range filtering would increase confidence.

Examining the balanced accuracy and sensitivity of the baseline models reveals the significance of integrating chemical and biological information for predicting cholestasis. This observation is consistent with findings from Kotsampasakou and Ecker,^12^ who combined physicochemical descriptors with hepatic transporter inhibition predictions for BSEP, BCRP, P-glycoprotein (P-gp), OATP1B1 and OATP1B3 to predict DIC. Additionally, Rodríguez-Belenguer et al.^28^ demonstrated that integrating mechanistic information from multiple molecular initiating events (MIEs) and QIVIVE models enhanced sensitivity, offering a more accurate depiction of cholestasis. Thus, incorporating biological information, such as liver-expressed pathways, improves sensitivity and MCC, likely due to the deeper mechanistic insights these pathways provide, or the broader matrix coverage compared to liver-expressed targets. To improve liver-expressed target coverage, four target prediction tools were used. However, performance comparisons showed that including all predicted liver-expressed targets improved MCC slightly but also increased specificity at the expense of sensitivity (Table 5), which is less desirable for toxicity prediction. This can be attributed to the fact, that incorporating predicted targets expanded the compound data set but also worsened class imbalance toward inactive compounds. Additionally, expanding the data set with more compounds and features resulted in cholestasis-positive compounds to be spread throughout the feature space rather than forming clusters (Figure 5). This expansion potentially reduces the effectiveness of identifying cholestasis effects.

An assessment of the predicted targets from the individual tools against cholestasis-related genes, listed in DisGeNet^52^ (version 24.1), revealed that the target prediction tools identified only a limited number of genes with a gene disease score (GDS) of 0.2 or higher for the cholestasis data set. Among the 149 genes in this category, ChEMBL Conformal Prediction predicted six, ChEMBL MNN identified 11, TargetNet predicted 13, and SEA predicted a higher number of 22 genes. Notably, the top five genes from Open Targets^61^ (https://platform.opentargets.org/disease/MONDO_0001751/associations) with the highest gene association scores for cholestasis – ABCB11, ATP8B1, ABCB4, USP53 and ZFYVE19 – along with the in our feature importance analysis highly ranked albumin, were not predicted by any of the target prediction tools, under the applied filters, for the cholestasis data set.

Incorporating all predicted targets increased the fingerprint length only marginally (Figure 4), while the number of interactions identified within liver-expressed targets and pathways substantially rose (Table 2). This demonstrates that integrating all predicted liver-expressed targets and pathways predominantly enhanced the matrix coverage of biological fingerprints rather than extending the fingerprint length. However, when comparing the matrix coverage of cholestasis-related genes between the baseline and prediction data sets, the coverage remains within the same range, even with target prediction (Table 3). The superior sensitivity of the baseline data set, coupled with the similar coverage of cholestasis-related genes, suggests that the target prediction process and the inclusion of potentially irrelevant pathways may have introduced noise into the prediction data sets. Therefore, accurately predicting DIC relies on comprehensive coverage of genes and pathways associated with cholestasis, which have not been extensively studied yet.

When integrating all descriptors, four PubChem bitvectors, two targets and four pathways emerged among the top ten descriptors (Table 11). Notably, the “Metabolism” pathway was highly ranked in the feature importance analysis. This higher-level pathway incorporates fatty acid metabolism and arachidonic acid metabolism, which are significant contributors in the feature importance analysis.^62^

Cholestasis is closely linked to impaired fatty acid metabolism in the liver.^63^ Since bile solutes are composed of approximately 12% fatty acids, variations in their composition can contribute to gallstone formation in the biliary tract.^64^ Furthermore, DIC is often characterized by inflammation, a response to liver injury.^65^ This inflammation is mediated by eicosanoids, which are the end products of the arachidonic acid metabolism pathway. Notably, nonsteroidal anti-inflammatory drugs (NSAIDs), which are a common cause of DIC, interact with this pathway. In a DILI prediction model, Füzi et al.^26^ also identified arachidonic acid and fatty acid metabolism as the top two descriptors in a feature importance analysis.

The sphingolipid de novo biosynthesis pathway, which produces sphingosin-1-phosphate (S1P) molecules, was also ranked high. Li et al.^66^ reported that in a mouse model of cholestasis-induced fibrosis, S1P and S1P3 receptor expression were upregulated in liver tissue. Jackson et al.^67^ further elucidated how bile acids and sphingolipids regulate hepatic lipid metabolism and noted the dysregulation of these pathways in conditions such as steatosis, inflammation and fibrosis in patients with Nonalcoholic Fatty Liver Disease (NAFLD). Li et al.^68^ underscored the critical role of sphingolipid metabolism and its disruption in hepatocellular death and liver injury, identifying this pathway as a sensitive readout and molecular marker of DILI. Therefore, studying sphingolipid metabolism in DIC could yield important mechanistic insights.

It is well-known that BSEP, also ranked very high, plays a crucial role in DIC by mediating the hepatic efflux of bile acids. Inhibition or dysfunction of BSEP can lead to accumulation of bile acids within the liver, resulting in cholestatic liver injury.^69^

In contrast, albumin, identified as the third most significant feature, has not been directly linked to cholestasis before. Albumin is synthesized in the liver and is the most prevalent plasma protein, comprising up to 60% of the total serum protein concentration.^70^ It plays a key role in modulating the pharmacokinetics of various drugs by reversibly binding to them.^71^ This binding influences the distribution, metabolism and elimination of drugs, thereby maintaining the pharmacologically active fraction of these substances in circulation.^72^ Reduced levels of serum albumin, or hypoalbuminemia, are frequently observed in hospitalized patients. Advanced cirrhosis, which represents the end stage of liver injury, is one example where this occurs.^73^ When albumin levels are diminished, highly plasma protein-bound drugs may cause toxicities.^74^ Analysis of drugs interacting with albumin in the cholestasis and DILI rank data sets revealed that a higher proportion of drugs associated with cholestasis and DILI fall into the high plasma protein binding class, compared to those in noncholestatic or no-DILI concern classes (Figure 6). Moreover, the displacement of one drug by another from plasma proteins, such as albumin, may result in toxic events.^75^

Given these findings, incorporating the plasma protein-bound fraction as an ADMET (absorption, distribution, metabolism, excretion, and toxicity) descriptor into the model seems reasonable. However, an initial analysis using Drug Bank pharmacology information^36^ revealed that plasma protein binding data is available for only 542 compounds of the baseline data set, substantially reducing its size. While predicting the plasma protein-bound fraction for the remaining compounds is an alternative, it would introduce additional uncertainty and could weaken the robustness of the models.

The analysis of structural characteristics reveals additional insights. Four bitvectors from the PubChem fingerprint were ranked among the top 10 descriptors. Notably, none of the identified PubChem bitvectors directly matches the 15 structural alerts of Firman et al.^29^ structural profiler. Instead, the PubChem bitvectors correspond to substructures within these chemotypes.

Bitvector 797 (CC1CC­(C)­CCC1) represents a complex SMARTS pattern corresponding to a six-membered carbon ring with two para-positioned methyl groups. This pattern is prevalent in the data set and is found in compounds with various indications, such as the antibiotic tetracycline. It is also present in steroid receptor modulators, including estrogenic and androgenic steroids. Estrogenic steroids are associated with cholestatic potential due to estrogen receptor binding, which leads to the downregulation of BSEP.^29,76^

In this regard, bitvector 590 (C–C:C–O–[#1]) represents a carbon atom bonded to a carbon–carbon double bond, which is further connected to an oxygen atom. This substructure is present in the stilbene derivative diethylstilbestrol and the phenolic ring A of estrogenic steroids such as estradiol (Table 12).

Another relevant PubChem bitvector is 439 (C­(−C)­(−N)­(=O)), which corresponds to an acetamide substructure present in beta-lactam antibiotics and the peptidomimetic core of ACE inhibitors.^29^ Notable examples include amoxicillin and ampicillin among beta-lactam antibiotics, and captopril and enalapril among ACE inhibitors. Firman et al.^29^ suggest that ACE inhibitors might induce cholestasis by increasing bradykinin levels.^77^ Beta-lactam antibiotics can cause cholestasis due to the intrinsic reactivity of their strained azetidinone substructure, which can interact with hepatocellular proteins, leading to haptenation and inflammation in hepatocytes.^29,78^

Bitvector 353 (C­(∼C)­(∼S)) falls into a category of PubChem fingerprints that evaluates nearest neighbors regardless of bond order. This bitvector aligns with the CS-bond chemotype identified by the structural profiler and is present in thiazolidinediones, which are labeled cholestasis-positive in the data set. Although the exact mechanism of liver damage by these drugs remains unclear, they induce PPARγ activation, which affects fatty acid and bile salt metabolism (https://www.ncbi.nlm.nih.gov/books/NBK551656/).^79,80^ Ongoing research in the AOP Wiki is investigating the relationship between PPARγ activation and intrahepatic cholestasis.^81^

Integrating probability range filtering with the consensus approach enhances decision-making in toxicity predictions. Consensus modeling, by merging multiple models, offers a robust prediction framework. The addition of probability range filtering refines this further, focusing on higher confidence results. Notably, the consensus model excluded more uncertain predicted compounds compared to the best performing model trained on undersampled data, indicating its capability to identify uncertainty (Table 9). Consequently, the probability range filtered consensus model showed a sensitivity of 0.80 for identifying cholestatic compounds in 10-fold cross-validation, while marking uncertain predictions. Although this approach results in a smaller number of classified compounds, it strengthens the reliability of risk assessments.

Using compounds without known liver-expressed targets served as a test case for compound prioritization. Compared to the best baseline model, this approach resulted in a decrease in MCC while maintaining stable sensitivity (Table 10). This finding suggests that pathways associated with known targets effectively capture the signal for classifying cholestasis-positive compounds, even when pathways are connected through predicted liver-expressed targets for untested compounds. However, the ability to correctly identify cholestasis-negative compounds based on known targets and their associated pathways remains limited and requires further refinement. A model with high sensitivity and low specificity may be overly cautious for compound prioritization, flagging many nontoxic compounds as toxic, which minimizes the risk of missing harmful compounds, but could lead to the unnecessary discarding of safe candidates.

## Conclusion

In this study, we developed a binary classification model to predict drug-induced cholestasis. This model integrates chemical data from PubChem substructure fingerprints with biological data, including experimentally measured liver-expressed compound-target and compound-pathway interactions, as well as nine hepatic transporter inhibition prediction models. Our best baseline model, which combined chemical and biological features, achieved performance on par with state-of-the-art models, as validated by 10-fold cross-validation and tested by a 20% holdout test set. Enhancing this model with predicted targets improved the MCC, though it resulted in reduced sensitivity.

The importance of integrating precise biological systemic information was further highlighted by our feature importance analysis. Specifically, pathways and targets emerged as significant features when combined with chemical data, aligning with existing literature. Notably, albumin was identified as a previously underexplored feature associated with cholestasis.

The consensus model demonstrated robust performance and increased decision-making confidence, with probability range filtering yielding the best results. This study represents an extension to current approaches by employing both traceable biological and chemical descriptors for drug-induced cholestasis prediction, offering an explainable and reliable method for this complex end point.

## Supplementary Material



## Data Availability

The KNIME workflow and data sets used in this study are publicly available in the PHAIDRA repository at https://phaidra.univie.ac.at/detail/o:2112256.

## Notes

(PDF) The Supporting Information is available free of charge at https://pubs.acs.org/doi/10.1021/acs.jcim.4c02363.

Threshold criteria for publicly available target prediction tools (Table S1), hyperparameters for machine learning algorithms (Table S2), performance statistics of all baseline models for 10-fold cross-validation (Table S3), and performance statistics of target prediction model with the best performing fingerprint for 10-fold cross validation (Table S4) (PDF)

## Abbreviations

NAM: new approach methodologies
DILI: drug-induced liver injury
DIC: drug-induced cholestasis
BSEP: bile salt export pump
MDR3: multidrug resistance protein 3
MRP3: multidrug resistance-associated protein 3
MRP4: multidrug resistance-associated protein 4
NTCP: sodium (Na^+^) taurocholate cotransporting polypeptide
Na^+^: Sodium
QSAR: quantitative structure–activity relationship
QIVIVE: quantitative in *vitro* and in *vivo* extrapolation
KNIME: Konstanz Information Miner
InChIKeys: IUPAC International Chemical Identifier Keys
SEA: similarity ensemble approach
MNN: multitask neural network
SMILES: simplified molecular input line entry system
kNN: k-nearest neighbors
RF: random forest
MATE1: multidrug and toxin extrusion protein 1
BCRP: breast cancer resistance protein
MDR1: multidrug resistance protein 1
OATP1B1: organic anion transporting polypeptide 1B1
OATP1B3: organic anion transporting polypeptide 1B3
OCT1: organic cation transporter 1
OCT2: organic cation transporter 2
ECFP4: extended connectivity fingerprint 4
SVR: support vector regression
LR: logistic regression
GB: gradient boosting
SMOTE: synthetic minority oversampling technique
US: undersampling
TP: true positives
FP: false positives
TN: true negatives
FN: false negatives
MCC: Matthews correlation coefficient
UMAP: uniform manifold approximation and projection
MIEs: molecular initiating events
GDS: gene disease score
S1P: sphingosin-1-phosphate
NSAIDs: nonsteroidal anti-inflammatory drugs
XGB: extreme gradient boosting
P-gp: P-glycoprotein
NAFLD: nonalcoholic fatty liver disease
