# Supplementary material for: Refining Drug-Induced Cholestasis Prediction: An Explainable Consensus Model Integrating Chemical and Biological Fingerprints
Source: J Chem Inf Model. 2025 May 27;65(11):5301–16. doi: 10.1021/acs.jcim.4c02363 (PMC12152943; doi:10.1021/acs.jcim.4c02363)
Supplement: Supplementary file 1 [file ci4c02363_si_001.pdf]

Supporting Information for Publication: “Refining Drug-Induced Cholestasis Prediction: An Explainable Consensus Model Integrating Chemical and Biological Fingerprints”

Palle S. Helmke and Gerhard F. Ecker

Department of Pharmaceutical Sciences, University of Vienna, 1090 Vienna, Austria

Corresponding author email: gerhard.f.ecker@univie.ac.at

Table S1. Threshold criteria for publicly available target prediction tools.

Target prediction tool	Threshold criterion	Threshold
ChEMBL multitask neural network	Probability	≥ 0.5
ChEMBL conformal prediction	Confidence intervals	70 % = active<br>80 % = active<br>90 % = active
TargetNet	Probability	≥ 0.5
Similarity ensemble approach	Maximum Tanimoto similarity	≥ 0.4
	pSEA (-log(p-value))	≥ 40

Table S2. Hyperparameter settings (KNIME default hyperparameters) and static random seeds for random forest and gradient boosting algorithms.

Algorithm	Number of levels (tree depth)	Number of models	Static random seed	Learning rate
Random forest	10	100	1711104736350	
Gradient boosting	10	100	1711104920607	0.1

Table S3. Performance statistics of all baseline models.

Fingerprint	Settings	Accuracy	MCC	Sensitivity	Specificity	Precision	Balanced accuracy
PubChem	Imb_GB	0.779	0.263	0.243	0.944	0.569	0.594
	Imb_RF	0.758	0.132	0.124	0.953	0.447	0.539
	SMOTE_GB	0.779	0.268	0.254	0.940	0.566	0.597
	SMOTE_RF	0.704	0.164	0.349	0.813	0.364	0.581
	US_RF	0.526	0.194	0.775	0.449	0.302	0.612
	**US_GB**	**0.573**	**0.233**	**0.757**	**0.516**	**0.325**	**0.637**
Target	Imb_GB	0.772	0.234	0.225	0.940	0.535	0.583
	Imb_RF	0.776	0.189	0.095	0.985	0.667	0.540
	SMOTE_GB	0.755	0.197	0.237	0.915	0.460	0.576
	SMOTE_RF	0.697	0.160	0.361	0.800	0.357	0.581
	**US_RF**	**0.602**	**0.261**	**0.751**	**0.556**	**0.342**	**0.654**
	US_GB	0.622	0.242	0.680	0.604	0.345	0.642
Pathway	Imb_GB	0.758	0.183	0.201	0.929	0.466	0.565

Fingerprint	Settings	Accuracy	MCC	Sensitivity	Specificity	Precision	Balanced accuracy
	Imb_RF	0.752	0.115	0.124	0.945	0.412	0.535
	SMOTE_GB	0.750	0.194	0.254	0.902	0.443	0.578
	SMOTE_RF	0.701	0.200	0.420	0.787	0.378	0.604
	**US_RF**	**0.563**	**0.223**	**0.757**	**0.504**	**0.319**	**0.631**
	US_GB	0.581	0.214	0.710	0.542	0.323	0.626
PubChem + Target	Imb_GB	0.783	0.256	0.189	0.965	0.627	0.577
	Imb_RF	0.773	0.195	0.136	0.969	0.575	0.553
	SMOTE_GB	0.776	0.255	0.243	0.940	0.554	0.592
	SMOTE_RF	0.732	0.201	0.331	0.855	0.412	0.593
	US_RF	0.579	0.236	0.751	0.525	0.327	0.638
	**US_GB**	**0.615**	**0.240**	**0.692**	**0.591**	**0.342**	**0.642**
PubChem + Pathway	Imb_GB	0.764	0.147	0.118	0.962	0.488	0.540
	Imb_RF	0.772	0.186	0.130	0.969	0.564	0.550
	SMOTE_GB	0.745	0.160	0.213	0.909	0.419	0.561
	SMOTE_RF	0.694	0.156	0.361	0.796	0.353	0.579
	US_RF	0.563	0.219	0.751	0.505	0.318	0.628
	**US_GB**	**0.605**	**0.286**	**0.787**	**0.549**	**0.349**	**0.668**
Target + Pathway	Imb_GB	0.762	0.180	0.178	0.942	0.484	0.560
	Imb_RF	0.771	0.187	0.142	0.964	0.545	0.553
	SMOTE_GB	0.751	0.176	0.219	0.915	0.440	0.567
	SMOTE_RF	0.690	0.157	0.373	0.787	0.350	0.580
	**US_RF**	**0.561**	**0.216**	**0.751**	**0.502**	**0.317**	**0.627**
	US_GB	0.580	0.202	0.692	0.545	0.319	0.619
PubChem + Transporter	Imb_GB	0.776	0.241	0.213	0.949	0.563	0.581
	Imb_RF	0.762	0.158	0.142	0.953	0.480	0.548
	SMOTE_GB	0.776	0.271	0.278	0.929	0.547	0.604
	SMOTE_RF	0.694	0.166	0.379	0.791	0.358	0.585
	**US_RF**	**0.540**	**0.220**	**0.793**	**0.462**	**0.312**	**0.628**
	US_GB	0.561	0.224	0.763	0.498	0.319	0.631
Target + Transporter	Imb_GB	0.780	0.262	0.231	0.949	0.582	0.590
	Imb_RF	0.773	0.174	0.095	0.982	0.615	0.539
	SMOTE_GB	0.761	0.214	0.243	0.920	0.482	0.582
	SMOTE_RF	0.712	0.194	0.379	0.815	0.386	0.597

Fingerprint	Settings	Accuracy	MCC	Sensitivity	Specificity	Precision	Balanced accuracy
	**US_RF**	**0.591**	**0.246**	**0.746**	**0.544**	**0.334**	**0.645**
	US_GB	0.598	0.204	0.663	0.578	0.326	0.621
Pathway + Transporter	Imb_GB	0.762	0.187	0.189	0.938	0.485	0.564
	Imb_RF	0.764	0.166	0.148	0.953	0.490	0.551
	SMOTE _GB	0.752	0.190	0.237	0.911	0.449	0.574
	SMOTE _RF	0.707	0.179	0.367	0.811	0.373	0.589
	**US_RF**	**0.569**	**0.243**	**0.781**	**0.504**	**0.326**	**0.643**
	US_GB	0.590	0.219	0.704	0.555	0.327	0.630
Target + Pathway + Transporter	Imb_GB	0.771	0.221	0.207	0.944	0.530	0.576
	Imb_RF	0.768	0.170	0.130	0.964	0.524	0.547
	SMOTE _GB	0.744	0.160	0.219	0.905	0.416	0.562
	SMOTE _RF	0.705	0.193	0.396	0.800	0.379	0.598
	**US_RF**	**0.572**	**0.235**	**0.763**	**0.513**	**0.325**	**0.638**
	US_GB	0.586	0.204	0.686	0.555	0.321	0.621
Pubchem + Target + Pathway	Imb_GB	0.777	0.217	0.148	0.971	0.610	0.560
	Imb_RF	0.764	0.147	0.118	0.962	0.488	0.540
	SMOTE _GB	0.764	0.170	0.154	0.951	0.491	0.553
	SMOTE _RF	0.726	0.198	0.343	0.844	0.403	0.594
	US_RF	0.573	0.226	0.746	0.520	0.323	0.633
	**US_GB**	**0.606**	**0.269**	**0.757**	**0.560**	**0.346**	**0.659**
Pubchem + Target + Transporter	Imb_GB	0.787	0.278	0.207	0.965	0.648	0.586
	Imb_RF	0.776	0.206	0.136	0.973	0.605	0.555
	SMOTE _GB	0.775	0.245	0.231	0.942	0.549	0.587
	SMOTE _RF	0.732	0.208	0.343	0.851	0.414	0.597
	**US_RF**	**0.583**	**0.240**	**0.751**	**0.531**	**0.330**	**0.641**
	US_GB	0.611	0.239	0.698	0.584	0.340	0.641
PubChem + Pathway + Transporter	Imb_GB	0.765	0.148	0.112	0.965	0.500	0.539
	Imb_RF	0.766	0.153	0.112	0.967	0.514	0.540
	SMOTE _GB	0.759	0.161	0.160	0.944	0.466	0.552
	SMOTE _RF	0.718	0.170	0.320	0.840	0.380	0.580
	US_RF	0.572	0.239	0.769	0.511	0.326	0.640
	**US_GB**	**0.605**	**0.289**	**0.793**	**0.547**	**0.350**	**0.670**
Pubchem + Target +	Imb_GB	0.773	0.192	0.130	0.971	0.579	0.551
	Imb_RF	0.769	0.157	0.101	0.975	0.548	0.538

Fingerprint	Settings	Accuracy	MCC	Sensitivity	Specificity	Precision	Balanced accuracy
Pathway + Transporter	SMOTE _GB	0.762	0.162	0.148	0.951	0.481	0.550
	SMOTE _RF	0.727	0.184	0.314	0.855	0.398	0.585
	US_RF	0.561	0.202	0.728	0.509	0.313	0.619
	**US_GB**	**0.611**	**0.260**	**0.734**	**0.573**	**0.345**	**0.654**

Table S4. Performance statistics of retrained target prediction models with the combined PubChem-pathway-transporter fingerprint for 10-fold cross-validation.

Model	Settings	Accuracy	MCC	Sensitivity	Specificity	Precision	Balanced accuracy
**+all**	Imb_GB	0.826	0.259	0.152	0.982	0.667	0.567
	Imb_RF	0.819	0.206	0.108	0.985	0.628	0.547
	SMOTE_GB	0.818	0.255	0.208	0.960	0.547	0.584
	SMOTE_RF	0.803	0.275	0.320	0.915	0.468	0.618
	US_RF	0.709	0.243	0.536	0.750	0.333	0.643
	**US_GB**	**0.720**	**0.301**	**0.616**	**0.744**	**0.359**	**0.680**
**+2-C**	Imb_GB	0.808	0.287	0.210	0.969	0.642	0.590
	Imb_RF	0.805	0.267	0.190	0.970	0.629	0.580
	SMOTE_GB	0.803	0.266	0.205	0.963	0.600	0.584
	SMOTE_RF	0.766	0.253	0.361	0.875	0.435	0.618
	US_RF	0.639	0.260	0.693	0.624	0.330	0.659
	**US_GB**	**0.659**	**0.280**	**0.688**	**0.651**	**0.346**	**0.670**
**+3-C**	Imb_GB	0.776	0.218	0.179	0.957	0.556	0.568
	Imb_RF	0.780	0.243	0.202	0.955	0.576	0.579
	SMOTE_GB	0.765	0.193	0.196	0.937	0.485	0.567
	SMOTE_RF	0.722	0.176	0.321	0.844	0.383	0.583
	US_RF	0.593	0.217	0.696	0.561	0.324	0.629
	**US_GB**	**0.619**	**0.247**	**0.696**	**0.595**	**0.342**	**0.646**
